# Nephroprotective Effect of *Asparagus africanus* Lam. Root Extract against Gentamicin-Induced Nephrotoxicity in Swiss Albino Mice

**DOI:** 10.1155/2022/8440019

**Published:** 2022-04-21

**Authors:** Welela Meka Kedir, Abebe Dukassa Dubiwak, Ebsa Tofik Ahmed

**Affiliations:** ^1^Department of Chemistry, College of Natural and Computational Sciences, Mettu University, Mettu, Ethiopia; ^2^Division of Medical Biochemistry, Department of Biomedical Sciences, Institute of Health Sciences, Jimma University, Jimma, Ethiopia; ^3^Department of Biology, College of Natural and Computational Sciences, Mettu University, Mettu, Ethiopia

## Abstract

The kidney is the organ most vulnerable to nephrotoxic drugs such as gentamicin. Nephrotoxicity is a rapid deterioration of kidney function due to various factors. Gentamicin causes nephrotoxicity, which was manifested by an increase in serum kidney biomarkers. *Asparagus africanus* is one of the ethnomedicinal plants used as traditional medicine for treating various ailments, including kidney disease in Ethiopian society. Thus, the aim of this study is to evaluate the nephroprotective effect of *A. africanus* root extract on gentamicin-induced nephrotoxicity. Using maceration techniques, 100 g of dried plant powder was extracted in 1 L of ethanol. The physicochemical screening of plant extracts revealed the presence of flavonoids, phenols, tannins, saponins, and steroids. The nephroprotective activity of *A. africanus* crude extract was evaluated on male Swiss albino mice. The crude ethanolic extract at 200 and 400 mg/kg doses showed strong nephroprotective effects by restoring biomarkers such as creatinine, uric acid, and blood urea nitrogen, which were damaged by gentamicin (*p* < 0.05) in a dose-dependent manner. The mice treated with higher doses (400 mg/kg) had a comparable nephroprotective effect compared to the positive control group (200 mg/kg silymarin; *p* > 0.05). The histopathology of the control group showed normal glomeruli, normal parenchyma, distal convoluted, and no tubular damage. The toxicant-induced group showed damage to glomeruli and inflammatory infiltration. Therefore, *A. africanus* root extract has a nephroprotective activity by retarding the gentamicin toxicity in male Swiss albino mice.

## 1. Introduction

Acute kidney injury is a syndrome characterized by the rapid loss of the kidney excretory function [1]. Acute renal failure refers to the sudden and usually reversible loss of renal function, which develops over a period of days or weeks [2]. Among the causes of acute renal failure, acute tubular necrosis, which occurs due to ischemia or nephrotoxins such as gentamicin (aminoglycoside), is most common, accounting for 85% of the incidences [3]. Nephrotoxicity has been reported in 1.7% to 58% of patients receiving aminoglycoside therapy [2, 3]. Gentamicin is an effective antibiotic that has been used worldwide for many years. While considered an essential medicine by the WHO, gentamicin can also lead to severe kidney damage [1, 2]. The recommended routes of administration of gentamicin are intravenous, intramuscular, intraperitoneal, or topical as it is not sufficiently absorbed by the intestinal tract [4, 5]. However, the potential clinical use of gentamicin is limited due to gentamicin-induced toxicity [4].

Gentamicin can cause tissue injury such as nephrotoxicity, ototoxicity [3, 4], and liver toxicity [5], possibly through the generation of free oxygen radicals. Nephrotoxicity of gentamicin arises due to its accumulation in renal cortical tubular epithelial cells [6]. Although the pathogenesis of gentamicin-induced acute kidney injury has been the focus of a large number of studies, the underlying mechanisms are not yet fully elucidated [7]. Recent studies suggest that gentamicin nephrotoxicity is a complex and multifaceted process in which gentamicin triggers cellular responses involving multiple pathways that culminate in renal damage and necrosis [8]. Several agents and strategies have been attempted to ameliorate gentamicin nephrotoxicity [7, 8], with the main focus on the use of various antioxidant agents, including the extracts from medicinal plants with antioxidant properties [9]. This biological activity may be attributed to its constituents obtained from plants, mainly phenolic compounds such as flavonoids. Flavonoids are well-known antioxidants possessing free radical scavenging and metal chelating activity [10]. Approximately 20% of all plants discovered on the planet have been subjected to pharmacological or biological testing, and a significant proportion of novel antibiotics are derived from natural or semisynthetic sources [11]. This fascinated the researchers to search out alternative sources of natural products with wide spectra of biological activities [12].

*Asparagus africanus*, locally called “Saritti” in (Afaan Oromo) and “Kestencha” in (Amharic), belongs to the family *Asparagaceae*, is a medicinal shrub valued for its medicinal properties. It is widely distributed throughout Africa, including Ethiopia, and parts of Europe, Asia, and Australia. *A*. *africanus*, with the common name “African *Asparagus*,” is a perennial shrub with stems up to 6 m high, growing between 700 and 3,800 m above sea level [13]. However, it is widely distributed and suitably grows higher, up to 6 m at an altitude range of 1,450–2,900 m [14]. *A. africanus* is known as a folk medicine in Ethiopia, and it is traditionally used for the management of kidney disease in the form of juice, tea, or soup [15]. Various studies also showed that *A. africanus* plant extract has different biological activities such as anti-bacterial, sexual impotency, gonorrhea, and syphilis [16]; hepatoprotective [17]; and antimalarial and insecticidal repellent properties [18]. In addition to this, *A*. *africanus* is used for pharmacological activities such as anti-diabetic [16, 19, 20], anti-protozoal [21], anti-inflammatory and analgesic [22], anti-fertility [19], anti-microbial [23], and anti-oxidant [24] activities. The genus *Asparagus* contains various phytochemicals such as tannins, alkaloids, terpenoids, steroids, and flavonoids that may cause a definite physiological action in the human body [25]. Chemical constituents of *A. africanus* plant extract revealed that the presence of several bioactive constituents, such as flavonoids, tannins, steroids, terpenoids, and saponin contained in *A. africanus* Lam. steroidal saponin, are the major active components of the genus *Asparagus* [26]. In this study, the nephroprotective activity of the root of *A. africanus* ethanolic extract was evaluated in two separate doses (200 and 400 mg/kg). In addition to that, the plant extract was compared with gentamicin (100 mg/kg) and silymarin (200 mg/kg) as negative and positive controls, respectively.

## 2. Hypothesis of the Study

### 2.1. Null Hypothesis

*A. africanus* ethanolic root extract has no significant nephroprotective effect on gentamicin-induced nephrotoxicity in Swiss albino mice.

### 2.2. Alternative Hypothesis

*A. africanus* ethanolic root extract has a significant nephroprotective effect on gentamicin-induced nephrotoxicity in Swiss albino mice.

## 3. Material and Methods

### 3.1. Study Area and Study Period

Extraction and phytochemical screening of plant-based products were carried out in the postgraduate Organic Chemistry Laboratory, College of Natural Sciences, Department of Chemistry, Jimma University, Jimma, Oromia Ethiopia. The nephroprotective effect of the plant extract was investigated at Jimma University's biotechnology laboratory, College of Agriculture and Veterinary Medicine. Serum analysis and kidney histopathology investigation were carried out at Jimma University Medical Center. The experiment was conducted from July 8 to August 7, 2020.

### 3.2. Chemicals and Drugs

Chemicals for extraction of phytochemical constituents such as petroleum ether (98%) and ethanol (99%) were used from LOBACHEMIE, Ltd. (India). Chemicals such as ketamine (100 mg/kg) and xylazine (12.5 mg/kg) were used for anesthesia. Gentamycin and silymarin (from Jimma University pharmaceutical laboratory) were used as negative and positive controls during the nephroprotective study, respectively. All the chemicals and reagents used in this study were analytical grade.

### 3.3. Apparatus and Instruments

Apparatus such as a rotary evaporator (Labo Rota 4000, Heidolph, USA) for solvent evaporation, oral gavage feeding for dose administration, a syringe for blood collection, a plastic cage for experimental animal grouping, and tissue slides for kidney histopathology analysis were used.

### 3.4. Plant Collection and Preparation

The root of *A*. *Africanus* was collected in August 2019 from southwestern Ethiopia, Bench-Maji Zone, Maji woreda, which is 568 km away from Addis Ababa. Identification of the plant species was made by the botanist, and a voucher specimen WM-01 was given and deposited at the Addis Ababa University Herbarium for further reference. The collected root of *A*. *Africanus* was washed, sliced, air-dried, and ground using an electrical grinder to improve subsequent extraction and penetration of the solvent into the cell.

### 3.5. Plant Extraction

The powdered plant material (100 g) was defeated with petroleum ether (0.5 L) for 24 h at room temperature to remove the fatty substance present in the sample by using a maceration technique. The solvent was filtered and then extracted with ethanol (1 L) for 24 h. The solvent extracts were filtered and concentrated using a rotary evaporator (Laborota-4000) at a temperature of 40°C with a speed of 90 rpm to have a solid consistency and dried by a freeze dryer (lyophilizer). Finally, residue extract was packed in air-tight glass bottles with proper labels and kept in a refrigerator at 4°C until used for the experiment [27].

### 3.6. Preliminary Phytochemical Screening

The ethanolic extract of *A*. *africanus* Lam. root was tested for alkaloids, terpenoids, flavonoids, phenol, steroid, quinones, saponin, tannin, and glycosides.

#### 3.6.1. Phenols (Ferric Chloride Test)

In a test tube, 0.2 g of plant extract was taken with 1 mL of water in a test tube, and two drops of Iron III chloride (FeCl_3_) were added. Blue, green, red, or purple color is a positive test [28].

#### 3.6.2. Saponins (Foam Test)

In a test tube, 0.1 g of plant extract was added and diluted with 20 mL distilled water and agitated in a graduated cylinder for 15 minutes. The formation of a 1 cm layer of foam indicates the presence of saponin [29].

#### 3.6.3. Alkaloids (Wagner's Test)

A plant extract (0.2 g) was added to a test tube, and three drops of Wagner's reagent (1.27 g of iodine and 2 g of potassium iodide in 100 mL of water) were added to the presence of alkaloids indicated by the formation of a reddish-brown precipitate [29].

#### 3.6.4. Tannins (Braymer's Test)

In a test tube, 1 mL 5% ferric chloride (FeCl_3_) was added to solvent-free 0.2 g extract. The presence of tannin is indicated by the formation bluish-black or greenish precipitate [28].

#### 3.6.5. Flavonoids (Sodium Hydroxide Test)

In a test tube, 0.2 g of test solution and a few drops of dilute sodium hydroxide (NaOH) were added; an intense yellow color was produced in the plant extract that becomes colorless with the addition of a few drops of dilute HCl. It indicates the presence of the flavonoid [28, 29].

#### 3.6.6. Terpenoids (Lieberman Bur Chard Test)

In a test tube, 0.2 g of test solution, 2 mL of chloroform, and 3 ml of concentrated sulfuric acid (H2SO4) were added to form a layer. The yellow color in a lower layer indicates the presence of terpenoids [30].

#### 3.6.7. Steroids (Salkowski Test)

A plant extract (0.2 g) dissolved in chloroform, in a different test tube, and a few drops of concentrated sulfuric acid were added to the solution, and dark pink/red color appeared indicating the presence of steroids [29, 30].

#### 3.6.8. Quinones

In a test tube, 0.2 g of plant extract was treated with a cone. HCl. Formation of a yellow precipitate (or coloration) confirms the presence of quinines [28].

#### 3.6.9. Glycoside

In a test tube, 0.1 g of the sample was dissolved in 5 mL of water, and then an aqueous 0.5 ml NaOH solution was added. Formation of yellow color indicates the presence of a glycoside [28, 30].

### 3.7. Experimental Animal

A total of 25 male Swiss albino mice with an average body weight of 35–40 g and an age of 8–10 weeks were obtained from the Tropical and Infectious Disease Research Center (TIDRC), Sokoru, Jimma, Ethiopia. Experimental animals were allowed to acclimate to laboratory conditions at the College of Agriculture and Veterinary Medicine, Jimma University, for 1 week before the experiment commenced. Standard food pellets (adlibitum) and tap water are provided in accordance with the OECD guidelines for the care and use of laboratory animals.

### 3.8. Experimental Design

Twenty-five male Swiss albino mice were divided randomly into five groups, each containing five per cage after one week of acclimatization. A permanent marker was used to write a number on each mouse's tail to distinguish it from the others in the group. The body weights of the mice were measured at the start of the experiment in order to adjust the extract dose administration.

### 3.9. Sample Size Determination

The sample size in this study was calculated using the resource equation approach. In this procedure, a value *E* is derived based on a sample size that has been determined. For optimal sample size, *E* should be in the range of 10 to 20. If *E* is less than 10, increasing the number of animals will improve the likelihood of obtaining more significant results, but if *E* is greater than 20, increasing the number of animals will not enhance the likelihood of obtaining significant results, and so the sample size should be reduced. Any sample size that keeps *E* between 10 and 20 should be regarded as sufficient [31, 32]. *E* = Total experimental animals − Total experimental groups = (5 × 5) − 5; 25 − 5 = 20. Hence, the sample size was 25, where a value “*E*” is measured, which is the degree of freedom of analysis of variance (ANOVA).

### 3.10. Experimental Protocol

The nephroprotective effect of *A*. *africanus* against gentamicin-induced nephrotoxicity was assessed in this investigation using a posttest-only control group design [33]. In this study, two separate doses of *A*. *africanus* root extract were evaluated based on the acute oral toxicity of the plant extract in the previously reported literature. The reported acute oral toxicity of *A*. *africanus* extract was safe up to 2,000 mg/kg [34]. The first dose was 200 mg/kg, which is 10% of the reported acute oral toxicity limit. The second dose was based on OECD guidelines, which require a constant scale-up of two, three, or four times the initial dose. Thus, 400 mg/kg was utilized as the second dose by twofold scaling up of the initial dose.

As shown in Table 1, the group I mice were fed a normal basal diet and received 1 mL/kg of distilled water orally for a period of 15 days. Group II mice were treated with 100 mg/kg of gentamicin injection orally for a period of 15 days [35–37], whereas group III mice were treated with silymarin 200 mg/kg and 100 mg/kg of gentamicin body mass for a period of 15 days, which was used for comparison with the treatment groups [38]. The group IV mice were induced with a 100 mg/kg gentamicin injection and *A*. *africanus* root extract at a dose of 200 mg/kg orally for a period of 15 days, and the group V mice were treated with a 100 mg/kg gentamicin injection and then administered *A*. *africanus* root extract at a dose of 400 mg/kg orally for a period of 15 days.

### 3.11. Sample Collection

On the 15^th^ day, the mice were fasted overnight and anesthetized with 100 mg/kg ketamine and 12.5 mg/kg xylazine injections. The mice were euthanized by cervical dislocation after 2–2.5 mL of blood collection through cardiac puncture [33, 39].

### 3.12. Biochemical Assays

Blood samples for the measurement of kidney biomarkers were drawn into serum separator tubes and left for 30 min at room temperature to clot. Serum was prepared by centrifugation of blood at 3,000 rpm for 10 min. The obtained serum was stored at −80°C until used for the biochemical assay. Serums were analyzed according to the standard principles and procedures outlined in the kit manufacturer's manual (SD Biosensor, Inc., Korea) [39].

### 3.13. Kidney Histopathology Examination

The kidney was rinsed with normal saline and then fixed in a 10% neutral buffered formalin solution, embedded in paraffin, and used for histopathological examination [33]. The sections were 2 *μ*m thick, deparaffinized, hydrated, and stained with hematoxylin and eosin. Senior pathologists were used a light microscope at a magnification of 400x to obtain microscopic observation of kidney tissue sections.

### 3.14. Data Analysis

Statistical analysis was carried out using SPSS version 25.0. The quantitative results were expressed as mean ± SD. One-way ANOVA and the Tukey post hoc tests were used for group comparisons. *p* < 0.05 is considered statistically significant. The qualitative histopathology examinations were performed by a senior pathologist.

### 3.15. Ethical Approval

The research was conducted after getting ethical approval from the Ethical Review Committee, College of Health Science, Jimma University. All experimental activities were carried out in accordance with the code of ethics for experimental animals, which complies with OECD guidelines.

## 4. Result

### 4.1. Result of Phytochemical Screening Test

After extraction, the percentage yield of the ethanolic extract of *A*. *Africanus* was 32%. The result of phytochemical screening of ethanolic extract of *A*. *Africanus* showed the presence of flavonoids, alkaloids, terpenoids, glycosides, saponins, tannin, phenol, and steroids (Table 2).

### 4.2. Nephroprotective Activity

In this study, the nephroprotective activity of *A*. *Africanus* extract was assessed by serum creatinine, blood urea nitrogen, and uric acid as well as histopathological analysis. The treatment root extract of A. Africanus showed a significant (*p* < 0.05) delay in the nephrotoxic effect of gentamicin on serum uric acid, blood urea nitrogen, and serum creatinine level (Table 3). The higher level of serum uric acid, blood urea nitrogen, and creatinine level in the blood or serum in the gentamicin-induced group is an indication of induced toxicity. However, when compared to group II, 400 mg/kg significantly reduced serum creatinine, uric acid, and BUN (*p* < 0.05). The overall effect of plant extract decreases those parameters compared to the toxicant-induced group in a dose-dependent manner (Table 3). In addition to that, the 200 mg/kg induced group decreased the serum uric acid and creatinine significantly compared to the toxicant control group (*p* < 0.05), but it reduced the serum blood urea nitrogen insignificantly (*p* > 0.05). The result of this study revealed that there was no significant difference between the normal control group and the GM coadministered with the silymarin-induced group (*p* > 0.05). Moreover, the higher dose of the plant extract (400 mg/kg) induced group decreased nonsignificantly the serum kidney biomarkers compared to the lower dose (200 mg/kg) induced group and the toxicant control. There was no significant difference between the 200 mg/kg and the toxicant control group (GM 100 mg/kg) on the serum blood urea nitrogen (*p* > 0.05), but serum creatinine and uric acid were significantly elevated in the GM-induced group compared to the 200 mg/kg induced group (*p* < 0.05).

### 4.3. Kidney Histopathological Findings

**Figure 1:**
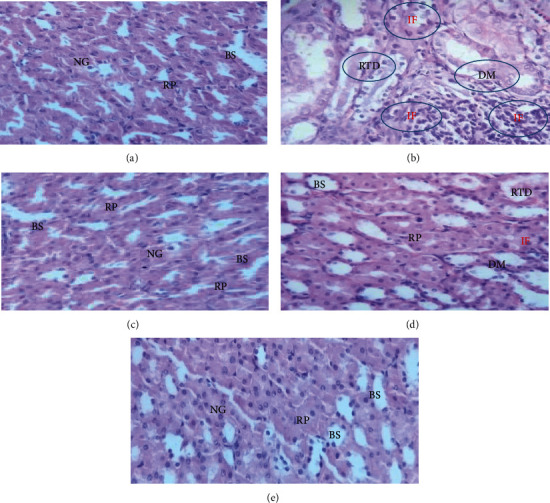
Photomicrograph of kidney section of animal (40x, stained with hematoxylin and eosin): group I (a), group II (b), group III (c), group IV (d), and group V (e). BS, Bowman's space; DM, damage of glomerulin; IF, inflammatory infiltration; NG, normal glomeruli; RP, renal parenchyma; and RTD, renal tubule degeneration.

The mice kidneys in the control group showed normal glomeruli with intact Bowman's capsule, normal proximal convoluted and distal convoluted, and no capillary congestion or hemorrhage (group 1). In the toxicant-induced group, undefined distortion of glomeruli, capillary congestion, hemorrhage, and apical blabbing were observed in the toxicant-induced group. Groups III (positive control), IV (200 mg/kg), and V (400 mg/kg) showed remarkably reduced capillary congestion, tubular damage, and glomerular distortion compared to the GM-treated group. The nephroprotective effect was found to be better with 400 mg/kg than with 200 mg/kg. The mice treated with 400 mg/kg did not show any abnormalities in biochemical parameters. The histopathological change was found to be correlated with biochemical change (Figure 1).

## 5. Discussion and Conclusion

### 5.1. Discussion

GM is known as an inducer of acute renal failure, which occurs in about 10% to 30% of patients [40]. The present study showed that GM-induced groups elevate the serum creatinine, blood urea nitrogen, and uric acid compared to the normal control group (*p* < 0.05). These indicate the mice induce toxicity and high-level kidney dysfunction. The new study's findings were in good accord with the prior study report from [3, 4], and it was claimed that injection of GM to rats during 14 days of treatment causes abnormal changes in kidney tissue such as a reduced cell in the glomeruli, loss of cellular tubular constituents, vascular congestion causing atrophy of epithelial cells, and distortions.

In this study, the decrease in serum creatinine, BUN, and uric acid in the 200 and 400 mg/kg induced groups compared to GM (*p* < 0.05) indicates the protective effect of plant extract. This may be due to the bioactive secondary metabolite such as flavonoid, tannin, and phenol present in the plant extract. The result is consistent with the previous study report from [37, 41] and stated that polyphenols and flavonoids reduce the nephrotoxicity of GM via an increase in the antioxidant enzymatic activity, a decrease in lipid peroxidation, scavenging free radicals, and improving the tissue architecture of the kidney. The serum creatinine and blood urea levels of 400 mg/kg of plant extract significantly decreased compared to 200 mg/kg (*p* < 0.05). This indicates the dose-dependent effect of plant extract and increase the concentration of the bioactive metabolite in the higher dose. During renal dysfunction, the kidney's clearance of creatinine (a no protein waste of creatinine phosphate metabolism) is reduced due to the reduction of glomerular filtration [42]. Not only creatinine but also serum uric acid and BUN were elevated due to the malfunction of the kidneys [43].

In this study, the elevation of serum creatinine, BUN, and uric acid was induced by GM and decreased by *A*. *africanus* extract. This effect may be associated with antioxidant activity and phytochemical and membrane stabilizing properties of plant extracts. A similar study reported that *A*. *africanus* plant extract has greater hepatoprotective and nephroprotective activity due to the phytochemical present in the plant extract [35]. In the same genus, *A*. *albus* L. leaves and *A. racemosus* root extract improved the structure of hepatic cells, confirming the hepatoprotective effect due to their free radical scavenging and antioxidant activity on account of their high polyphenols, flavonoids, and condensed tannin [44, 45]. In general, administration of 200 and 400 mg/kg had a beneficial effect on the kidney treated with GM. This was evidenced by a significantly decreased serum creatinine, BUN, and uric acid in the *A*. *africanus* treated group when compared to the GM-induced group.

### 5.2. Conclusion

The root of *A*. *africanus* ethanolic extract at a dose of 400 mg/kg has comparable nephroprotective activity to the positive control silymarin, which is further confirmed by histopathological examination of the kidney and its serum biomarkers. In general, mice treated with *A*. *africanus* ethanolic extract possess potential protective activity against gentamycin-induced nephrotoxicity. It is due to the secondary metabolite present in the plant extract. However, further investigations are required into antioxidant enzymes or markers of lipid peroxidation in renal tissues or serum with various solvent extracts of plants.

## Figures and Tables

**Table 1:** Experimental animals grouping and treatment protocol for a period of 15^th^ day.

Group	Category	Dose administration
I	Normal control	Normal basal diet + 1 mL/kg of distilled water
**II**	Negative control	100 mg/kg IP gentamicin + 1 mL/kg of distilled water
**III**	Positive control	100 mg/kg IP gentamicin + silymarin 200 mg/kg
**IV**	Treatment one	100 mg/kg IP gentamicin +200 mg/kg extract
**V**	Treatment two	100 mg/kg IP gentamicin +400 mg/kg extract

**Table 2:** The preliminary phytochemical screening of ethanolic extract of the root of *A*. *africanus*.

Phytochemicals	Result	Phytochemicals	Results
Flavonoid	+	Terpenoid	−
Alkaloid	+	Saponins	+
Terpenoids	+	Tannin	+
Quinone	−	Phenol	+
Glycosides	+	Steroid	+

“+” stands for the presence of phytochemicals and “−” stands for the absence of phytochemicals.

**Table 3:** The effects of *A*. *africanus* root extract on gentamicin-induced nephrotoxic mice.

Groups	Creatinine (mg/dl)	Uric acid (mg/dl)	BUN (mg/dl)
Group I (normal control)	0.62 ± 0.01	1.72 ± 0.04	22.71 ± 0.90
Group II (GM 100 mg/kg)	1.88 ± 0.01^a^	3.84 ± 0.05^a^	42.72 ± 0.69^a^
Group III (GM 100 mg/kg + sylim100 mg/kg)	0.67 ± 0.02^b^	1.66 ± 0.06^b^	23.66 ± 0.36^b^
Group IV (extract 200 mg/kg)	1.78 ± 0.01^b^	3.28 ± 0.07^b^	42.36 ± 0.64
Group V (extract 400 mg/kg)	0.79 ± 0.01^b^	1.67 ± 0.02^b^	23.08 ± 0.38^b^

The results were expressed as mean ± SD. GM = gentamicin. The results were analyzed by analysis of variance (ANOVA) followed by the Tukey multiple comparison test; *n* = 5. a = significant difference compared to group I, b = significant difference compared to group II, and *p* < 0.05 was considered statistically significant.

## Data Availability

The data used to support the findings of this study are available from the corresponding author upon request.
